# Shining Light on Autophagy in Skin Pigmentation and Pigmentary Disorders

**DOI:** 10.3390/cells11192999

**Published:** 2022-09-26

**Authors:** Daniela Kovacs, Giorgia Cardinali, Mauro Picardo, Emanuela Bastonini

**Affiliations:** Cutaneous Physiopathology and Integrated Center of Metabolomics Research, San Gallicano Dermatological Institute (IRCCS), 00144 Rome, Italy

**Keywords:** autophagy, skin pigmentation, AMPK, mTOR, hypopigmentation, hyperpigmentation, vitiligo, senile lentigo, melasma

## Abstract

Autophagy is a vital process for cell survival and it preserves homeostasis by recycling or disassembling unnecessary or dysfunctional cellular constituents. Autophagy ameliorates skin integrity, regulating epidermal differentiation and constitutive pigmentation. It induces melanogenesis and contributes to skin color through melanosome turnover. Autophagy activity is involved in skin phenotypic plasticity and cell function maintenance and, if altered, it concurs to the onset and/or progression of hypopigmentary and hyperpigmentary disorders. Overexpression of autophagy exerts a protective role against the intrinsic metabolic stress occurring in vitiligo skin, while its dysfunction has been linked to the tuberous sclerosis complex hypopigmentation. Again, autophagy impairment reduces melanosome degradation by concurring to pigment accumulation characterizing senile lentigo and melasma. Here we provide an updated review that describes recent findings on the crucial role of autophagy in skin pigmentation, thus revealing the complex interplay among melanocyte biology, skin environment and autophagy. Hence, targeting this process may also represent a promising strategy for treating pigmentary disorders.

## 1. Introduction

The fascinating variation in skin color associated with ethnic heterogeneity and the color-changing ability ranging from invertebrates to vertebrates species involves the production and the removal of pigment organelles, as well as recycling pathways that connect the two phases. Such pigmentation plasticity characterized by pigmentation–depigmentation–repigmentation cycles is sustained, at the cellular level, by the turnover of pigments deposited within specialized intracellular organelles belonging to the lysosome-related organelle (LRO) family, of which mammalian melanosomes are the prototype [1,2].

In human skin, melanosomes represent the melanocyte compartment specifically deputed to the synthesis of melanin and its transfer to the neighboring keratinocytes. During stages I and II of development, melanosomes are not pigmented and melanin synthesis begins at stage III until stage IV of fully melanized melanosomes [3]. The biosynthesis of melanin is a complex process that starts with the oxidation of L-tyrosine to dopaquinone by the rate-limiting enzyme activity of tyrosinase (TYR) in cooperation with other tyrosinase-related melanogenic enzymes (tyrosinase-related TRP-1 and TRP-2) to produce two types of pigments: dark-brown eumelanin and reddish-yellow pheomelanin [4]. The transport of melanosomes from the perinuclear area to the cell periphery involves complex molecular machinery that regulates the movement on microtubules and actin filaments [5]. The subsequent transfer of melanosomes from melanocytes to keratinocytes is a crucial event to ensure effective photoprotection by the dispersion of the pigment in the skin. This process occurs through different mechanisms triggered by UV-induced melanosome-transfer-related pathways [6,7]. Upon UV exposure, melanogenesis is stimulated through the release of paracrine factors by keratinocytes and fibroblasts such as α-melanocyte-stimulating hormone (α-MSH), stem cell factor (SCF), endothelin-1 (ET-1) and prostaglandins. The multiple pathways activated by these factors converge on the microphthalmia-associated transcription factor (MITF), the master regulator of melanogenic enzymes and melanocyte proliferation, differentiation and survival [8,9]. The human skin color is determined by the content/composition of melanin and the distribution and packaging of mature melanosomes in the epidermis. Melanosomes from dark skin are larger and dispersed throughout the cytosol of keratinocytes. Vice versa, in light skin, such organelles are smaller and aggregated in clusters [10]. Moreover, melanosomes are more abundant in African American keratinocytes than in Caucasian ones [11] and light keratinocytes degrade melanosomes more efficiently in comparison to dark cells [12,13].

The intriguing aspect that has recently emerged is the substantial contribution of the autophagy process in each phase of the physiological melanosome turnover (biosynthesis, maturation and degradation) [14]. Autophagy is an evolutionarily conserved and highly orchestrated self-cannibalization process that guarantees intracellular nutrient balance by selective recycling of cytoplasmic components within lysosomes, thus providing energy and metabolic precursors as well as removing damaged proteins and/or organelles [15,16,17]. Eukaryotic cells continuously deal with a dynamic environment which, over a specific threshold, may result in multiple types of stressors such as changes in nutrient, energy and oxygen sources and in protein folding/organelles status. Hence, autophagy is a cell’s major adaptive/survival strategy serving as a homeostatic mechanism at the subcellular level to respond to environmental changes and survive against injuries. On the other hand, massive autophagy under pathological conditions can lead to excessive degradation of cellular contents and trigger a form of cell death known as “autophagic cell death” (CDA) [18] that was first elegantly described in insects, occurring during their metamorphosis [19]. Consequently, dysfunction in autophagy-related processes causes severe human pathologies [20,21].

Based on different types of cargo and various modes of cargo delivery, autophagy is defined according to three main canonical classifications: macroautophagy, microautophagy and chaperone-mediated autophagy. Over the past two decades, the molecular mechanisms underlying the types of autophagy have been characterized in detail.

Macroautophagy (from here onward referred to as autophagy) is the most comprehensively studied and the autophagosome, which is encircled by a double-lipid bilayer that engulfs cargo targeted for being removed, is its morphological hallmark. In physiological conditions, canonical autophagic responses can be subdivided into five phases: initiation, nucleation, elongation and substrate selection, autophagosome–lysosome fusion and lysosomal substrate degradation [22]. All the biochemical reactions, ranging from the generation of autophagosomes to the delivery of cargo to lysosomes for degradation, involve at least 100 different proteins. Among those, more than 40, arise from a set of evolutionarily conserved genes initially identified in yeast, having been classified as “autophagy-related” (*ATGs*) genes [23]. Their loss-of-function mutation seems not to be tolerated and the embryonic loss of ATG7 results in neonatal death in mice and causes death in adulthood after its inducible whole-body knock-out, thus highlighting “the importance of being autophagic” [24]. Post-translational protein modifications such as phosphorylation, ubiquitination and acetylation play a central role in coordinating the activity of ATG proteins [25].

MAP1LC3B/LC3B (microtubule-associated protein 1 light chain 3 b), a small 18-kDa protein of the LC3 family, which is processed and modified by conjugation to phosphatidylethanolamine (PE) to generate a lapidated form of LC3 (LC3-II), which is attached to both faces of the autophagosome membrane, but is ultimately removed from the outer membrane, is present during the entirety of the autophagic process [26]. Many roles have been assigned to this protein, including autophagosome elongation, closure, maturation and cargo recognition [27].

The initial trigger of autophagy comes from a variety of stressors, such as starvation, hypoxia, oxidative stress and damaged/misfolded proteins. All these signal converge in the activation of Unc-51-like autophagy activating kinase complex (ULK). Two distinct kinases, belonging to intersecting pathways, are key players in the orchestration of autophagy control through phosphorylation of ULK1 [28]. Adenosine monophosphate (AMP)-activated protein kinase (AMPK), which is activated by reduced energy charge through the increase in AMP/ATP ratio, positively regulates autophagy resulting in the activation of the autophagy-initiation complex including FIP200, ULK1 and ATG13 [29]. On the contrary, the mammalian target of rapamycin (mTOR), which is a stress-sensitive kinase complex that controls the intracellular metabolic state by shifting catabolism to growth-promoting anabolism, is a suppressor of autophagy [30]. More specifically, under starvation or rapamycin treatment, mTOR is dissociated from the initiation complex, and ATG13 and ULK1 are partially dephosphorylated, thus inducing autophagy [31].

Autophagy is also regulated by the Beclin 1–interacting complex, consisting of Beclin 1, class III phosphatidylinositol-3-kinase (PIK3C3, or VPS34) and ATG14L. Activation of this complex generates phosphatidylinositol-3-phosphate (PI3P), which is involved in the autophagosomal membrane nucleation [32]. Different cargo-selective proteins, also known as autophagy receptors, which bind or may be an integral part of the cargo, recognize and link the ubiquitinated substrate to the autophagy machinery [33]. After the autophagosome fuses with lysosomes, cargo degradation is operated by luminal acidification and lysosomal hydrolases producing basic building blocks of complex macromolecules, such as amino acids, which are subsequently recycled back into the cytoplasm for reuse.

Two other forms of autophagy have been described: microautophagy by which cytoplasmatic entities destinated for degradation are directly obtained by invagination of the lysosomal membrane [34] and chaperone-mediated autophagy (CMA) whose peculiarity is that neither vesicles nor membrane invaginations are required for cargo delivery to lysosomes [35]. CMA only degrades soluble proteins through a protein-translocation complex (chaperone) by which they reach the surface of the lysosomes. Once there, the substrate proteins unfold and cross the lysosomal membrane.

**Figure 1:**
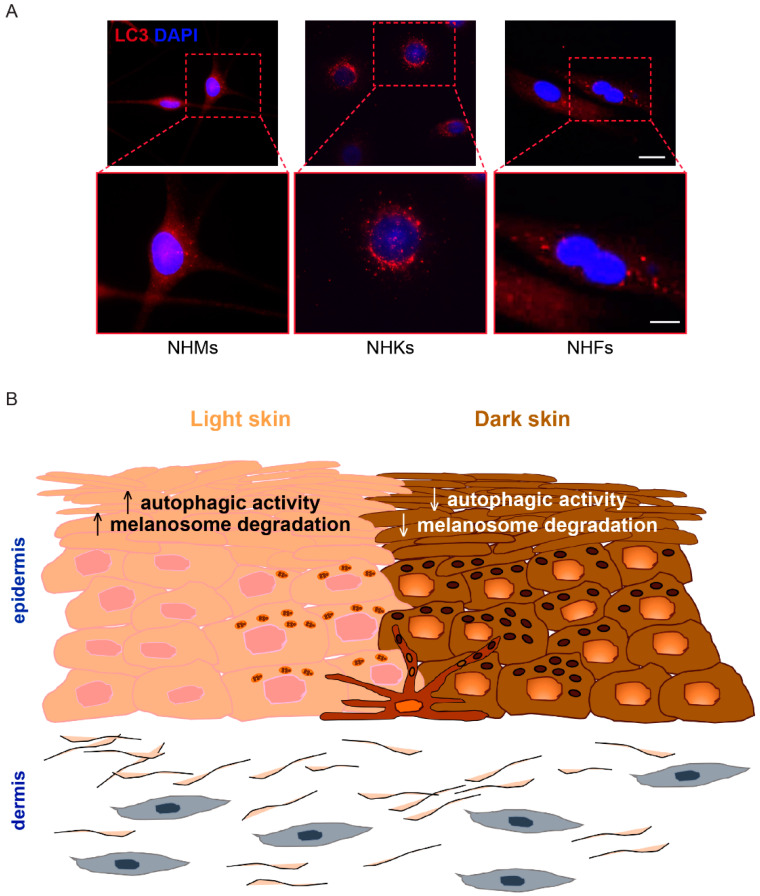
Skin color and autophagy. (**A**) Immunofluorescence analysis was performed on primary cultures of normal human melanocytes (NHMs), normal human keratinocytes (NHKs) and normal human fibroblasts (NHFs) using an antibody (red signal) directed against the autophagy marker light chain 3 (LC3I/II). Nuclei are counterstained with DAPI (blue signal). Scale bars: 20 μm; enlarged view of the boxed area: 10 μm. (**B**) Autophagic activity is involved in skin color variation by regulating melanosome degradation.

In the skin, behind the crucial intracellular quality control in keratinocytes, melanocytes and fibroblasts [36], autophagy is involved in cell remodeling during development and differentiation processes. Representative images of LC3 expression in melanocytes, keratinocytes and dermal fibroblasts are shown in Figure 1A. The interaction between autophagy and apoptosis is also an important part of epidermal homeostasis [37,38].

Activation of autophagy is known to induce melanogenesis [3] and regulates the life cycle of melanosomes in keratinocytes and melanocytes [39], thus indicating the involvement of this process in the different skin pigmentation phenotypes [13,40]. Rapamycin-induced autophagy significantly increased melanin index, tyrosinase activity and the expression of MITF in Melan-a melanocytes [40]. Vice versa, LC3 depletion suppressed αMSH-mediated melanogenesis by reducing cAMP response element-binding protein (CREB) phosphorylation, MITF expression and subsequent rapamycin-induced melanosome formation. Interestingly, under stress conditions, the inhibition of the mTOR pathway stimulates the nuclear translocation of MITF. Within the nucleus, MITF is implicated in the transcription of autophagy genes [41]. Overexpression of autophagy protects melanocytes from oxidative stress-induced apoptosis. On the contrary, suppression of ATG7-dependent autophagy inhibits proliferation and results in premature senescence [42].

To date, the role of LC3 in regulating pigmentation is complex and still controversial. Some studies reported that knock-down of LC3 markedly attenuated melanin synthesis and that LC3 co-localized with the melanosome markers Pmel17 in mature melanosomes and MART1 in sunlight-exposed melanocytic nevi [40]. On the contrary, Zhang et al., showed that LC3-deficient melanocytes synthesized melanin regularly [43]. Interestingly, as the name microtubule-associated protein 1 light chain 3 b evokes, LC3 is involved in melanosome movement on microtubules in melanocytes, thus assuming a role in organelle trafficking pathways [44].

The complex role that autophagy plays in melanocyte biology and pigmentation persists even when melanocytes undergo malignant transformation. As for other cancers, in the dynamic and intricate scenario of melanoma, autophagy exerts dual and opposite actions depending on the stage of the disease. During the onset of the tumor, it acts as a tumor suppressor mechanism to remove long-lived and/or dysfunctional cellular constituents and maintain genetic stability. Meanwhile, as melanoma progresses and metastasizes, autophagy is upregulated to guarantee nutrient demands and survival in the unfavorable tumor microenvironment, acting as adaptive tumor-promoting machinery. Current knowledge hence supports how complex appears the interplay between the dynamism of the autophagic process and the plasticity and heterogeneity characterizing melanomas [45,46,47].

In keratinocytes, several different and seemingly contradictory biological roles are covered by autophagy. Indeed, such process has been described as a mechanism strictly related to differentiation [48], survival against UV-induced damage [49,50,51] and melanosome degradation. It is well known that keratinocytes derived from light skin degrade melanosomes more rapidly than keratinocytes derived from dark skin [52]. However, the link between the different phototypes and autophagic activity is still being investigated. Murase et al. [13] demonstrated a higher autophagic activity in Caucasian keratinocytes than in those derived from African American skin, thus indicating the involvement of the process in modulating skin color variations (Figure 1B). Interestingly, Gosselin and co-workers demonstrated that apoptotic markers do not appear concurrently with senescence in keratinocytes. Instead, senescent cells die by autophagic programmed cell death that targets primary vital cell components, such as nuclei and mitochondria [53]. These findings suggest that the upregulation of autophagy may represent a compensatory mechanism to ensure the death of apoptosis-resistant keratinocytes. Moreover, as cornification of keratinocytes involves the degradation of intracellular constituents, the autophagy-mediated control of terminal differentiation may concur to the maintenance of epidermal homeostasis [54]. Therefore, it is likely that the precise role of autophagy may be dependent on the stage of keratinocyte differentiation. Interestingly, the ultrastructural analysis revealed acanthosis, hyperkeratosis, abnormal hair growth and overall retardation of granular layer differentiation in the skin grafts from the ATG7-deficient mice [55]. Belleudi et al. [56] proposed that the dynamic proliferation/differentiation cell-state transition may be also regulated by the fibroblast growth factor 7 (FGF7) through a fine modulation of the autophagy process. To further explore the role of autophagy in keratinocytes, Qiang and co-workers recently demonstrated that keratinocytes from *ATG5/7*-knockout mice showed a decrease in proliferation and differentiation levels in the wound neoepidermis [57].

Several studies have revealed that not only the epidermal–melanin unit but even dermal fibroblasts play an effective role in regulating skin pigmentation by acting on melanocytes directly and/or indirectly through the secretion of a large number of cytokines, proteins and growth factors [58]. Moreover, dermal fibroblasts represent long-lived differentiated cells that constantly undergo intrinsic and extrinsic damaging effects, thus representing physiological indicators for human aging [59]. Autophagy functionally contributes to decreasing the rate of the aging process. However, the activity of autophagy declines during aging and aged dermal fibroblasts show an impaired capacity to remove oxidized and misfolded proteins as well as DNA damage. Treatment of young dermal fibroblasts with lysosomal inhibitors to mimic the dysfunctional autophagic activity characterizing aged fibroblasts changed the content of type I procollagen, hyaluronan and elastin and produced a breakdown of collagen fibrils [60]. These data suggest that autophagy impairment in aged fibroblasts may concur with the decline in dermal structural integrity and skin fragility. Recently, autophagy has been reported to also regulate extracellular matrix (ECM) by modulating the expression of MMPs and the MMP-mediated collagen degradation in skin fibroblasts [61].

The role of autophagy in epidermal stem cells is still unexplored. Shi et al., found that mutations in the C-terminal domain of Gsdma3, which was originally identified in association with the hair-loss phenotype in mouse mutants and is expressed in epidermal stem cells, induces autophagy to likely balance the reservoir of stemness and the level of differentiated epidermal cells [62].

**Figure 2:**
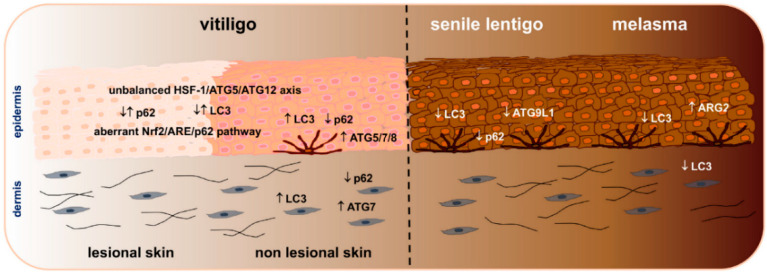
Autophagy dysregulation in hypo- and hyper-pigmented disorders: summary of autophagic markers known to be up- and/or down-modulated in epidermal and dermal compartments of lesional and non-lesional vitiligo, senile lentigo and melasma skin.

It is increasingly evident that autophagy is a core pathway involved in a multitude of biological mechanisms that regulate skin homeostasis, including pigmentation. Dysregulations in autophagic activity have been observed in both hypo- and hyper-pigmentary disorders (Figure 2).

## 2. Autophagy Deregulation in Hypopigmentary Disorders

### 2.1. Vitiligo

Vitiligo is the most common acquired cutaneous depigmenting disorder characterized by the gradual loss of melanocytes driven by multiple mechanisms that finally lead to the destruction of melanocytes by cytotoxic CD8+ cells [63,64]. The precise role of autophagy in vitiligo has yet to be defined. Although contrasting data seem to emerge, some evidence suggests the involvement of this process in the pathogenesis of the disease. Vitiligo cells are highly susceptible to oxidative stress and display impaired antioxidant defense machinery, including the nuclear erythroid 2-related factor 2 (Nrf2)/antioxidant response element (ARE) pathway and its downstream antioxidant and detoxifying enzymes [65]. The altered responses of vitiligo melanocytes to stressors have been linked to a reduced autophagic flux due to the dysfunctions of Nrf2-p62 pathway. In normal cells, under oxidative stress, Nrf2 dissociates from its negative regulator Keap1 and translocates to the nucleus where it activates the transcription of several detoxifying enzymes and the autophagic adaptor p62. Hence, the impaired activation of Nrf2/p62 pathway demonstrated in vitiligo cells causes an autophagy defect and higher vulnerability to oxidative injuries [66]. In response to oxidative stress, vitiligo melanocytes also display a reduced gene expression of *ATG5* and *ATG12*, whose lower levels are caused by a deficiency in the expression of their transcription factor HSF-1. Since defects in HSF-1 are responsible for intracellular ROS accumulation and increased apoptosis upon exposure to oxidative stressors, the unbalance of the HSF-1-ATG5/ATG12 axis has been suggested to be crucially involved in the impaired activation of autophagy and the oxidative stress-susceptibility of vitiligo cells [67].

As the autophagy-defective melanocytes displayed features resembling premature senescence, accompanied by aberrant Nrf2 signaling, redox unbalance and increased lipid oxidation, Zhang et al., hypothesized a model shared by both autophagy-deficient cells and vitiligo cells, thus pointing to autophagy as a potential factor acting on vitiligo pathogenesis [43]. The link between autophagy and vitiligo derives also from studies focused on the mRNAs profile of autophagy-related genes. Differences in the expression of several autophagy-related genes have been detected in leukocytes from non-segmental vitiligo (NSV) patients in comparison to those from healthy individuals [68]. More recently, RNA sequencing on tissue samples collected from stable NSV patients revealed the inhibition of autophagy in lesional skin, as also confirmed by the decreased ratio of LC3II/LC3 associated with increased p62 expression [69]. A possible association between polymorphisms of the autophagy-promoting gene *UVRAG* (ultraviolet radiation resistance-associated gene) and increased susceptibility to NSV has been suggested in the Korean population [70], once again connecting vitiligo pathogenesis to autophagy dysregulations.

Conversely, the induction of autophagy has been also reported in vitiligo. Yu et al., found a higher expression of LC3 and ATG5 associated with lower expression of p62 in lesional skin with respect to control. Interestingly, stable vitiligo lesions showed increased autophagy in comparison to active lesions, leading the authors to hypothesize that the induction of autophagy may exert a protective and/or restorative function to counteract vitiligo progression [71]. In line with these data, Raam et al., demonstrated the presence of augmented autophagic vacuoles in the residual melanocytes as well as keratinocytes of lesional areas in comparison to non-lesional areas and the control skin [72].

The involvement of autophagy has also been considered in relation to the inflammatory milieu characterizing vitiligo skin. In this context, it has been demonstrated that the pro-inflammatory cytokine TNF-α, highly expressed in vitiligo, favors the rise of intracellular ROS, elevates *IL-6* and *ICAM 1* expression, affects melanocyte viability and reduces melanogenesis. Interestingly, these effects were accompanied by the early (after 12 h) induction of autophagy, as assessed by the increased levels of *ATG12* and *BECN1* transcripts. However, in the presence of prolonged TNF-α exposure (48 h), cell survival shifted to apoptosis, thus indicating a functional connection between autophagy and melanocyte destruction [73]. Among the inflammatory mediators involved in vitiligo pathogenesis, IL-17 is reported to be elevated in the serum and lesional skin of patients and it is positively correlated to the extent of depigmentation [74,75]. Zhou et al., evidenced that IL-17 inhibits melanogenesis and induces mitochondrial dysfunction as well as ROS production in melanocytes. The rise of intracellular stress due to functional impairment of mitochondria was accompanied by increased expression of Beclin 1, LC3 and ATG5. Parallel to autophagy, IL-17 treatment also stimulated the activation of caspase 8 and the expression of pro-apoptotic proteins, hence establishing that autophagy is involved in IL-17 mediated apoptosis via stress generation in melanocytes [76]. It has also been recently shown that lipopolysaccharide (LPS) inhibits melanin synthesis by activating autophagy in the vitiligo melanocyte cell line PIG3V, thus suggesting a novel insight into the pathogenesis of the disease linked to the role of LPS in microbial-induced changes of pigmentation [77].

Our group has proved that melanocytes and fibroblasts isolated from the non-lesional skin of vitiligo patients display increased expression of autophagic markers. We showed that the induction of autophagy functions as a protective mechanism to counterbalance the intrinsic metabolic impairment characterizing vitiligo. Accordingly, we found that the energy metabolism enhancer, N-acetyl cysteine, reduces the expression of autophagy markers and concurrently increases the levels of intracellular ATP. Vice versa, the treatment with the pro-oxidant tertbutyl hydroperoxide (t-BHP) able to decrease ATP production and thus mimicking metabolic alteration, induced the overexpression of autophagic markers in normal melanocytes [78]. We also demonstrated that the inhibition of autophagy exacerbates the degenerative features of vitiligo cells, worsening the appearance of a senescent associated phenotype, highlighted by the increased expression of p53, its target genes *GADD45*, *p21* and *p16*. Hence, autophagy results as an attempt to counteract the intrinsic metabolic stress and the degenerative process of vitiligo cells in normally pigmented skin, where melanocytes and fibroblasts are already inclined to premature senescence [78] (Figure 2).

The different scenarios emerging from the variable role attributed to autophagy in vitiligo mirror the complexity and multiplicity of the mechanisms involved in determining melanocyte loss, which include genetic, autoimmune-mediated, oxidative and metabolic dysregulations. The increased or decreased induction of autophagy may be thus dependent on a wide array of variabilities. Autophagy levels may be modulated according to the active or stable status of the disease. It may be hypothesized that in the progressive phase, the entity of the immune responses may exacerbate the local redox and metabolic dysfunctions and promote a broad release of inflammatory mediators, all conditions known to impact autophagy. Likewise, the intrinsic functional features and the surrounding milieu of different skin areas, e.g., lesional, perilesional or non-lesional sites, may variably affect the autophagic flux. Once the depigmentation becomes evident, it might be speculated that the compensatory/protective function of autophagy demonstrated in non-lesional cells [78] can be exacerbated to such an extent, to shift from a pro-survival to a pro-autophagic cell death outcome. Alternatively, the increased autophagy found in the non-lesional areas, as well as in stable versus active vitiligo lesions [71], could gradually progress to the exhaustion of its activity, making cells unable to counteract the pro-oxidant insults. This autophagy-related impairment, in association with intrinsic metabolic defects and an unfavorable microenvironment, may therefore contribute to melanocyte destruction and favor the onset and progression of the depigmented macules. However, what might be the decisive factors orienting the process in one direction or another are yet to be determined.

### 2.2. Tuberous Sclerosis Complex

Defects in pigmentation linked to autophagy dysfunction characterize the tuberous sclerosis complex, an autosomal dominant disorder resulting from a pathogenic variant of *TSC1* or *TSC2* genes, which are responsible for the constitutive activation of the negative regulator for autophagy-mTOR. The disease presents multiple hamartomas with epilepsy, neuropsychiatric symptoms and focal hypopigmentation at birth or early during infancy, manifestations overall recognized to be ascribed to mTOR hyperactivation [79]. Employing immortalized human primary melanocytes and the highly pigmented human melanoma cell line SK-Mel-30, Cao et al., showed that suppression of *TSC1* or *TSC2* expression reduces pigmentation, decreases tyrosinase activity and lowers the levels of MITF and its downstream pigment-related target genes: *TYR*, *TYRP1*, *PMEL* and *DCT*. The decrease in melanogenesis results from enhanced mTORC1 activity, as assessed by the restoration of pigmentation in response to the mTORC1 inhibitor rapamycin. The mechanism by which pigmentation is modulated by mTORC1 involves activation of GSK3β, phosphorylation and decreased nuclear levels of β-catenin, which reduce the expression of MITF and its target genes. In agreement with these data, the occurrence of TSC complex loss has been confirmed in melanocyte cultures collected from hypomelanotic macules of patients with TSC [80]. Autophagic dysregulation in melanocytes of patients with TSC has been also demonstrated by Yang et al., who observed mTOR-hyperactivation in association with the reduced amount of pigment in hypopigmented macules of TSC patients. Cultures of *TSC2*-knockdown (*TSC2*-KD) primary melanocytes appeared less pigmented and presented autophagy dysregulations, with increased expression of LC3 and accumulation of substrates for autophagic degradation. Again, similar features were detected in melanocytes of hypopigmented macules of patients with TSC. Furthermore, in *TSC2*-KD melanocytes, the inhibition of autophagy accelerates depigmentation, whereas mTOR-dependent and mTOR-independent autophagy enhancers abrogate pigment reduction, providing that autophagic impairment is responsible for the TSC depigmentation [81].

### 2.3. Pigmentary Mosaicism

Pigmentary mosaicism refers to hypo and/or hyperpigmentation patterns resulting from skin cells’ genetic heterogeneity, frequently associated to neurocutaneous involvement [82]. Pigmentation-related signs are variable in size and number and localized along Blashko’s lines. Autophagy of immature melanosomes in keratinocytes associated with melanosomes depletion has been described as a prominent aspect in a case report of pigmentary mosaicism characterized by hypochromic lesions, suggesting a possible role of autophagy [83]. Additionally, hypopigmentation in mosaic mTOR pathogenic variants has been reported to be possibly due to a defect in melanogenesis attributed to mTOR complex hyperactivation, as shown for TSC [84].

## 3. Autophagy Deregulation in Hyperpigmentary Disorders

### 3.1. Senile Lentigo

Human skin undergoes chronological aging due to intrinsic factors (time, genetics and hormones) and environmental aging linked to exposure to extrinsic stimuli (air pollution, cigarette smoke, nutritional factors, stress and UV irradiation). The aging process affects skin functionality, regenerative capacity and causes pigmentary alterations, such as hyper melanosis, characterized by increased melanogenesis and reduced melanosome degradation. Interestingly, autophagy defects are associated with skin aging and autophagic activity decreases with age when some pigmentary abnormalities emerge [3,12,13,40,61,85,86,87,88]. Several studies evaluated whether an impairment of autophagy, by decreasing melanosome degradation, may underlie the onset of pigmentary disorders [12,13,14,40,89]. Senile lentigo (SL) is a benign tumor occurring in aged people and it is characterized by the presence of light brown to black pigmented spots in chronically UV-exposed skin areas. Histological analysis shows several melanocytes and a great amount of melanin in the pigmented lesions. Melanocytes are hyperfunctional and keratinocytes display altered expression of some differentiation markers [90,91,92,93]. Murase et al., demonstrated signs of premature aging and decreased expression of LC3, p62, and ATG9L1 in SL lesional areas in comparison to non lesional ones [12]. These alterations are associated with enhanced melanogenesis and aberrant epidermal differentiation, thus evidencing a relationship between hyperpigmentation and autophagic deficit. In the same study, the autophagy process has been also evaluated in skin samples obtained from the inner, upper and elbow regions of African American (AA) and Caucasian individuals. In AA individuals, the elbows represent a sun-exposed area characterized by small hyperpigmented spots often associated with severe dehydration. Similar to SL, the elbow skin of AA shows melanin deposition up to the stratum corneum and epidermal dehydration due to altered keratinocyte differentiation and proliferation. Again, these features are associated with a significant decrease in LC3, p62, and ATG levels and, in addition, an increase in mTORC1. In ex vivo skin models, the treatment with the inhibitor of mTORC1 Torin 1 restored autophagy deficit and improved hyperpigmentation and epidermal differentiation (Figure 2).

### 3.2. Melasma

Melasma is a chronic acquired hypermelanosis mainly occurring in women with high phototype (III-IV) during fertility age and principally involving the photo-exposed skin areas such as the face. This pigmentary disorder is characterized by the increased amount of melanin due to the higher number and retention of mature melanosomes, hyper-functional melanocytes, dermal alterations and sebocytes activation associated with photoaging and inflammation [94,95]. The etiopathogenesis of melasma is still not completely understood. Although photoaging has a crucial role in the onset of this hypermelanosis, other factors besides UV are certainly involved in favoring the aging process and the accumulation of senescent cells. Senescent keratinocytes (from late passages) show age-related pigmentation [96,97] and impaired autophagy [60,98,99]. Kim et al., investigated the role of miR-1299 on arginase-2 (ARG2) regulation in hyper and normally pigmented skin from melasma patients [100]. The downregulation of miR-1299 is associated with the overexpression of ARG2, which reduces autophagy by inducing cell senescence. Therefore, the senescence process in keratinocytes would be the cause of autophagy deficit which, in turn, results in an increased pigmentation due to the reduced degradation of melanosomes. Cavalcante Esposito et al., compared the expression of LC3 in lesional and perilesional skin samples from women affected by facial melasma [101]. The study showed that melanocytes (basal and pendulum) and upper dermis fibroblasts displayed a deficit in autophagy compared to unaffected skin. Similar to hypopigmented lesions, a link between autophagy and inflammation has also been shown for hyperpigmented disorders. Autophagy-defective melanocytes express high levels of pro-inflammatory cytokines and chemokine ligands (*CXCL1, 2, 10* and *12*) that, in turn, are involved in pigmentary disorders associated with a higher expression of metalloproteinases 3 and 13 [102] (Figure 2).

The approaches to treat hyper melanosis are mainly focused on the inhibition of MC1R, the regulation of MITF expression and tyrosinase activity [103,104]. However, tyrosinase inhibitors lead to disappointing clinical results due to their toxicity/carcinogenicity and low efficacy [104,105,106]. More recently, tranexamic acid (trans-4-amino-methylcyclohexanecarboxylic acid, TXA) has been employed to treat melasma [107] and UV-induced hyperpigmentation [108,109]. TXA is an anti-fibrinolytic agent acting by the inhibition of plasminogen activator (PA). Kim et al., demonstrated that TXA can reduce melanin content by decreasing tyrosinase activity and the expression of melanogenesis-related enzymes (tyrosinase, TRP-1 and TRP-2) in melanocytes and melanoma cells [110]. Interestingly, Cho et al., demonstrated that the treatment with TXA increases the expression of the autophagy-related proteins (MAPKs, ERKs, Beclin 1, ATG12 and LC3) and decreases the mTOR complex in B16-F1 melanoma cells [111]. In parallel, the expression of MITF, tyrosinase and TRP1/2 are reduced. Therefore, TXA can reduce melanin content through the activation of the autophagic system, representing a good candidate for melasma. Kim et al., showed that PTPD-12, an autophagy-inducing peptide, increases LC3 expression and Beclin 1 phosphorylation, favoring melanosome degradation without affecting MITF expression and melanosome biogenesis [39]. Moreover, topical application of PTPD-12 on ex vivo and 3D skin models induces a lightening effect by decreasing melanin content, without affecting melanocyte survival.

The development of autophagy-inducing treatments able to promote melanosome degradation without interfering with melanogenesis can be considered the most promising in terms of efficacy and safety [112].

## 4. Conclusions

Autophagy represents an intriguing player in regulating constitutive pigmentation and, if altered, the development and progression of pigmentary disorders. Studies focused on a deeper knowledge of the complex interactions among autophagic machinery, melanocyte biology and skin environment may contribute to highlighting novel decisive elements in the etiopathogenesis of pigmentary disorders. Furthermore, innovative therapeutic strategies targeting autophagy and/or its regulating factors may result as effective to prevent the onset and/or undermine the progression of pigmentary disorders.

## Data Availability

Not applicable.

## References

[1] Delevoye C., Marks M.S., Raposo G. (2019). Lysosome-related organelles as functional adaptations of the endolysosomal system. Curr. Opin. Cell Biol..

[2] Ullate-Agote A., Burgelin I., Debry A., Langrez C., Montange F., Peraldi R., Daraspe J., Kaessmann H., Milinkovitch M.C., Athanasia C. (2020). Genome mapping of a LYST mutation in corn snakes indicates that vertebrate chromatophore vesicles are lysosome-related organelles. Proc. Natl. Acad. Sci. USA.

[3] Raposo G., Marks M.S. (2007). Melanosomes-dark organelles enlighten endosomal membrane transport. Nat. Rev. Mol. Cell Biol..

[4] D’Alba L., Shawkey M.D. (2019). Melanosomes: Biogenesis, Properties, and Evolution of an Ancient Organelle. Physiol. Rev..

[5] Tian X., Cui Z., Liu S., Zhou J., Cui R. (2021). Melanosome transport and regulation in development and disease. Pharmacol. Ther..

[6] Tadokoro R., Takahashi Y. (2017). Intercellular transfer of organelles during body pigmentation. Curr. Opin. Genet. Dev..

[7] Wu X., Hammer J.A. (2014). Melanosome transfer: It is best to give and receive. Curr. Opin. Cell Biol..

[8] Vachtenheim J., Borovanský J. (2010). “Transcription physiology” of pigment formation in melanocytes: Central role of MITF. Exp. Dermatol..

[9] Liu J.J., Fisher D.E. (2010). Lighting a path to pigmentation: Mechanisms of MITF induction by UV. Pigment. Cell Melanoma Res..

[10] Minwalla L., Zhao Y., Le Poole I.C., Wickett R.R., Boissy R.E. (2001). Keratinocytes play a role in regulating distribution patterns of recipient melanosomes in vitro. J. Investig. Dermatol..

[11] Montagna W., Carlisle K., Beaverton M.S. (1991). The architecture of black and white facial skin. J. Am. Acad. Dermatol..

[12] Murase D., Kusaka-Kikushima A., Hachiya A., Fullenkamp R., Stepp A., Imai A., Ueno M., Kawabata K., Takahashi Y., Hase T. (2020). Autophagy Declines with Premature Skin Aging resulting in Dynamic Alterations in Skin Pigmentation and Epidermal Differentiation. Int. J. Mol. Sci..

[13] Murase D., Hachiya A., Takano K., Hicks R., Visscher M.O., Kitahara T., Hase T., Takema Y., Yoshimori T. (2013). Autophagy has a significant role in determining skin color by regulating melanosome degradation in keratinocytes. J. Investig. Dermatol..

[14] Zhu W., Zhao Z., Cheng B. (2020). The role of autophagy in skin pigmentation. Eur. J. Dermatol..

[15] Klionsky D.J., Abdel-Aziz A.K., Abdelfatah S., Abdellatif M., Abdoli A., Abel S., Abeliovich H., Abildgaard M.H., Abudu Y.P., Acevedo-Arozena A. (2021). Guidelines for the use and interpretation of assays for monitoring autophagy (4th edition). Autophagy.

[16] Mizushima N. (2007). Autophagy: Process and function. Genes Dev..

[17] Levine B., Klionsky D.J. (2004). Development by self-digestion: Molecular mechanisms and biological functions of autophagy. Dev. Cell.

[18] Debnath J., Baehreccke E.H., Kroemer G. (2005). Does autophagy contribute to cell death?. Autophagy.

[19] Schwartz L.M. (1992). Insect muscle as a model for programmed cell death. J. Neurobiol..

[20] Lei Y., Klionsky D.J. (2021). The Emerging Roles of Autophagy in Human Diseases. Biomedicines.

[21] Klionsky D.J., Petroni G., Amaravadi R.K., Baehrecke E.H., Ballabio A., Boya P., Bravo-San Pedro J.M., Cadwell K., Cecconi F., Choi A.M.K. (2021). Autophagy in major human diseases. EMBO J..

[22] Galluzzi L., Bravo-San Pedro J.M., Levine B., Green D.R., Kroemer G. (2017). Pharmacological modulation of autophagy: Therapeutic potential and persisting obstacles. Nat. Rev. Drug Discov..

[23] Ohsumi Y. (2014). Historical landmarks of autophagy research. Cell Res..

[24] Ganley Y. (2021). The importance of being autophagic. N. Engl. J. Med..

[25] Levine B., Kroemer G. (2019). Biological function of autophagy genes: A disease perspective. Cell.

[26] Weidberg H., Shvets E., Shpilka T., Shimron F., Shinder V., Elazar Z. (2010). LC3 and GATE-16/GABARAP subfamilies are both essential yet act differently in autophagosome biogenesis. EMBO J..

[27] Wild P., McEwan D.G., Dikic I. (2014). The LC3 interactome at a glance. J. Cell Sci..

[28] Kim J., Kundu M., Viollet B., Guan K.L. (2011). AMPK and mTOR regulate autophagy through direct phosphorylation of Ulk1. Nat. Cell Biol..

[29] Garcia D., Shaw R.J. (2017). AMPK: Mechanisms of Cellular Energy Sensing and Restoration of Metabolic Balance. Mol. Cell..

[30] Saxton R.A., Sabatini D.M. (2017). mTOR signaling in growth, metabolism and disease. Cell.

[31] Jung C.H., Jun C.B., Ro S.H., Kim Y.M., Otto N.M., Cao J., Kundu M., Kim D.H. (2009). ULK-Atg13-FIP200 complexes mediate mTOR signaling to the autophagy machinery. Mol. Biol. Cell.

[32] Choi A.M.K., Ryter S.W., Levine B. (2013). Autophagy in human health and disease. N. Engl. J. Med..

[33] Gatica D., Lahiri V., Klionsky D.J. (2018). Cargo recognition and degradation by selective autophagy. Nat. Cell Biol..

[34] Li W.W., Li J., Bao J.K. (2012). Microautophagy: Lesser-known self-eating. Cell Mol. Life Sci..

[35] Kaushik S., Cuervo A.M. (2012). Chaperone-mediated autophagy: A unique way to enter the lysosome world. Trends Cell Biol..

[36] Sukseree S., Eckhart L., Tschachler E., Watanapokasin R. (2013). Autophagy in epithelial homeostasis and defence. Front Biosci. (Elite Ed.).

[37] Chikh A., Sanzà P., Raimondi C., Akinduro O., Warnes G., Chiorino G., Byrne C., Harwood C.A., Bergamaschi D. (2014). iASPP is a novel autophagy inhibitor in keratinocytes. J. Cell Sci..

[38] Chikh A., Matin R.N., Senatore V., Hufbauer M., Lavery D., Raimondi C., Ostano P., Mello-Grand M., Ghimenti C., Bahta A. (2011). iASPP/p63 autoregulatory feedback loop is required for the homeostasis of stratified epithelia. EMBO J..

[39] Kim J.Y., Kim J., Ahn Y., Lee E.J., Hwang S., Almurayshid A., Park K., Chung H.J., Kim H.J., Lee S.H. (2020). Autophagy induction can regulate skin pigmentation by causing melanosome degradation in keratinocytes and melanocytes. Pigment. Cell Melanoma Res..

[40] Yun W.J., Kim E.Y., Park J.E., Jo S.Y., Bang S.H., Chang E.J., Chang S.E. (2016). Microtubule-associated protein light chain 3 is involved in melanogenesis via regulation of MITF expression in melanocytes. Sci. Rep..

[41] Ozturk D.G., Kocak A., Akcay K., Kinoglu E., Kara Y., Buyuk H., Kazan H., Gozuacic D. (2019). MITF-MIR211 axis is a novel autophagy amplifier system during cellular stress. Autophagy.

[42] Qiao Z., Xu Z., Xiao Q., Yang Y., Ying J., Xiang L., Zhang C. (2020). Dysfunction of ATG7-dependent autophagy dysregulates the antioxidant response and contributes to oxidative stress-induced biological impairments in human epidermal melanocytes. Cell Death Discov..

[43] Zhang C.F., Gruber F., Ni C., Mildner M., Koenig U., Karner S., Barresi C., Rossiter H., Narzt M.S., Nagelreiter I.M. (2015). Suppression of autophagy dysregulates the antioxidant response and causes premature senescence of melanocytes. J. Investig. Dermatol..

[44] Ramkumar A., Murthy D., Raja D.A., Singh A., Krishnan A., Khanna S., Vats A., Thukral L., Sharma P., Sivasubbu S. (2017). Classical autophagy proteins LC3B and ATG4B facilitate melanosome movement on cytoskeletal tracks. Autophagy.

[45] Corazzari M., Fimia G.M., Lovat P., Piacentini M. (2013). Why is autophagy important for melanoma? Molecular mechanisms and therapeutic implications. Semin. Cancer Biol..

[46] Rahmati M., Ebrahim S., Hashemi S., Motamedi M., Moosavi M.A. (2020). New insights on the role of autophagy in the pathogenesis and treatment of melanoma. Mol. Biol. Rep..

[47] Di Leo L., Bodemeyer V., De Zio D. (2020). The Complex Role of Autophagy in Melanoma Evolution: New Perspectives From Mouse Models. Front. Oncol..

[48] Aymard E., Barruche V., Naves T., Bordes S., Closs B., Verdier M., Ratinaud M.H. (2011). Autophagy in human keratinocytes: An early step of the differentiation?. Exp. Dermatol..

[49] Song X., Narzt M.S., Nagelreiter I.M., Hohensinner P., Terlecki-Zaniewicz L., Tschachler E., Grillari J., Gruber F. (2017). Autophagy deficient keratinocytes display increased DNA damage, senescence and aberrant lipid composition after oxidative stress in vitro and in vivo. Redox Biol..

[50] Qiang L., Wu C., Ming M., Viollet B., He Y.Y. (2013). Autophagy controls p38 activation to promote cell survival under genotoxic stress. J. Biol. Chem..

[51] Zhao Y., Zhang C.F., Rossiter H., Eckhart L., König U., Karner S., Mildner M., Bochkov V.N., Tschachler E., Gruber F. (2013). Autophagy is induced by UVA and promotes removal of oxidized phospholipids and protein aggregates in epidermal keratinocytes. J. Investig. Dermatol..

[52] Ebanks J.P., Koshoffer A., Wickett R.R., Schwemberger S., Babcock G., Hakozaki T., Boissy R.E. (2011). Epidermal keratinocytes from light vs. dark skin exhibit differential degradation of melanosomes. J. Investig. Dermatol..

[53] Gosselin K., Deruy E., Martien S., Vercamer C., Bouali F., Dujardin T., Slomianny C., Houel-Renault L., Chelli F., De Launoit Y. (2009). Senescent keratinocytes die by autophagic programmed cell death. Am. J. Pathol..

[54] Mahanty S., Dakappa S.S., Shariff R., Patel S., Swamy M.M., Majumdar A., Setty S.R.G. (2019). Keratinocyte differentiation promotes ER stress-dependent lysosome biogenesis. Cell Death Dis..

[55] Yoshihara N., Ueno T., Takagi A., Oliva Trejo J.A., Haruna K., Suga Y., Komatsu M., Tanaka K., Ikeda S. (2015). The significant role of autophagy in the granular layer in normal skin differentiation and hair growth. Arch. Dermatol. Res..

[56] Belleudi F., Purpura V., Caputo S., Torrisi M.R. (2014). FGF7/KGF regulates autophagy in keratinocytes: A novel dual role in the induction of both assembly and turnover of autophagosomes. Autophagy.

[57] Qiang L., Yang S., Cui Y.H., He Y.Y. (2021). Keratinocyte autophagy enables the activation of keratinocytes and fibroblasts and facilitates wound healing. Autophagy.

[58] Kapoor R., Dhatwalia S.K., Kumar R., Rani S., Parsad D. (2020). Emerging role of dermal compartment in skin pigmentation: Comprehensive review. J. Eur. Acad. Dermatol. Venereol..

[59] Tigges J., Krutmann J., Fritsche E., Haendeler J., Schaal H., Fischer J.W., Kalfalah F., Reinke H., Reifenberger G., Stühler K. (2014). The hallmarks of fibroblast ageing. Mech. Ageing Dev..

[60] Tashiro K., Shishido M., Fujimoto K., Hirota Y., Yo K., Gomi T., Tanaka Y. (2014). Age-related disruption of autophagy in dermal fibroblasts modulates extracellular matrix components. Biochem. Biophys. Res. Commun..

[61] Jeong D., Qomaladewi N.P., Lee J., Park S.H., Cho J.Y. (2020). The Role of Autophagy in Skin Fibroblasts, Keratinocytes, Melanocytes, and Epidermal Stem Cells. J. Investig. Dermatol..

[62] Shi P., Tang A., Xian L., Hou S., Zou D., Lv Y., Huang Z., Wang Q., Song A., Lin Z. (2015). Loss of conserved Gsdma3 self-regulation causes autophagy and cell death. Biochem. J..

[63] Seneschal J., Boniface K., D’Arino A., Picardo M. (2021). An update on Vitiligo pathogenesis. Pigment. Cell Melanoma Res..

[64] Bergqvist C., Ezzedine K. (2021). Vitiligo: A focus on pathogenesis and its therapeutic implications. J. Dermatol..

[65] Jian Z., Li K., Song P., Zhu G., Zhu L., Cui T., Liu B., Tang L., Wang X., Wang G. (2014). Impaired activation of the Nrf2-ARE signaling pathway undermines H2O2-induced oxidative stress response: A possible mechanism for melanocyte degeneration in vitiligo. J. Investig. Dermatol..

[66] He Y., Li S., Zhang W., Dai W., Cui T., Wang G., Gao T., Li C. (2017). Dysregulated autophagy increased melanocyte sensitivity to H2O2-induced oxidative stress in vitiligo. Sci. Rep..

[67] Cui T., Wang Y., Song P., Yi X., Chen J., Yang Y., Wang H., Kang P., Guo S., Liu L. (2022). HSF1-Dependent Autophagy Activation Contributes to the Survival of Melanocytes Under Oxidative Stress in Vitiligo. J. Investig. Dermatol..

[68] Wang P., Li Y., Nie H., Zhang X., Shao Q., Hou X., Xu W., Hong W., Xu A. (2016). The changes of gene expression profiling between segmental vitiligo, generalized vitiligo and healthy individual. J. Dermatol. Sci..

[69] Yang Y., Wu X., Lu X., Wang C., Xiang L., Zhang C. (2022). Identification and Validation of Autophagy-Related Genes in Vitiligo. Cells.

[70] Jeong T.J., Shin M.K., Uhm Y.K., Kim H.J., Chung J.H., Lee M.H. (2010). Association of UVRAG polymorphisms with susceptibility to non-segmental vitiligo in a Korean sample. Exp. Dermatol..

[71] Yu H., Lin X., Huang Y., Cheng H., Seifert O. (2021). The Difference in Expression of Autophagy-Related Proteins in Lesional and Perilesional Skin in Adult Patients with Active and Stable Generalized Vitiligo-A Cross-Sectional Pilot Study. Indian J. Dermatol..

[72] Raam L., Kaleviste E., Šunina M., Vaher H., Saare M., Prans E., Pihlap M., Abram K., Karelson M., Peterson P. (2018). Lymphoid Stress Surveillance Response Contributes to Vitiligo Pathogenesis. Front. Immunol..

[73] Singh M., Mansuri M.S., Kadam A., Palit S.P., Dwivedi M., Laddha N.C., Begum R. (2021). Tumor Necrosis Factor-alpha affects melanocyte survival and melanin synthesis via multiple pathways in vitiligo. Cytokine.

[74] Bassiouny D.A., Shaker O. (2011). Role of interleukin-17 in the pathogenesis of vitiligo. Clin. Exp. Dermatol..

[75] Singh R.K., Lee K.M., Vujkovic-Cvijin I., Ucmak D., Farahnik B., Abrouk M., Nakamura M., Zhu T.H., Bhutani T., Wei M. (2016). The role of IL-17 in vitiligo: A review. Autoimmun. Rev..

[76] Zhou J., An X., Dong J., Wang Y., Zhong H., Duan L., Ling J., Ping F., Shang J. (2018). IL-17 induces cellular stress microenvironment of melanocytes to promote autophagic cell apoptosis in vitiligo. FASEB J..

[77] Jun S.L., Sun J., Huo X., Feng Q., Li Y., Xie X., Geng S. (2022). Lipopolysaccharide reduces melanin synthesis in vitiligo melanocytes by regulating autophagy. Exp. Dermatol..

[78] Bastonini E., Kovacs D., Raffa S., Delle Macchie M., Pacifico A., Iacovelli P., Torrisi M.R., Picardo M. (2021). A protective role for autophagy in vitiligo. Cell Death Dis..

[79] Wataya-Kaneda M. (2015). Mammalian target of rapamycin and tuberous sclerosis complex. J. Dermatol. Sci..

[80] Cao J., Tyburczy M.E., Moss J., Darling T.N., Widlund H.R., Kwiatkowski D.J. (2017). Tuberous sclerosis complex inactivation disrupts melanogenesis via mTORC1 activation. J. Clin. Investig..

[81] Yang F., Yang L., Wataya-Kaneda M., Hasegawa J., Yoshimori T., Tanemura A., Tsuruta D., Katayama I. (2018). Dysregulation of autophagy in melanocytes contributes to hypopigmented macules in tuberous sclerosis complex. J. Dermatol. Sci..

[82] Schaffer J.V. (2022). Pigmentary mosaicism. Clin. Dermatol..

[83] Devillers C., Quatresooz P., Hermanns-Lê T., Szepetiuk G., Lemaire R., Piérard-Franchimont C., Piérard G.E. (2011). Hypomelanosis of Ito: Pigmentary mosaicism with immature melanosome in keratinocytes. Int. J. Dermatol..

[84] Carmignac V., Mignot C., Blanchard E., Kuentz P., Aubriot-Lorton M.H., Parker V.E.R., Sorlin A., Fraitag S., Courcet J.B., Duffourd Y. (2021). Clinical spectrum of MTOR-related hypomelanosis of Ito with neurodevelopmental abnormalities. Genet. Med..

[85] Zhong Y., Wang Q.J., Li X., Yan Y., Backer J.M., Chait B.T., Heintz N., Yue Z. (2009). Distinct regulation of autophagic activity by Atg14L and Rubicon associated with Beclin 1-phosphatidylinositol-3-kinase complex. Nat. Cell Biol..

[86] Ho H., Ganesan A.K. (2011). The pleiotropic roles of autophagy regulators in melanogenesis. Pigment. Cell Melanoma Res..

[87] Kim E.S., Shin J.H., Seok S.H., Kim J.B., Chang H., Park S.J., Jo Y.K., Choi E.S., Park J.S., Yeom M.H. (2013). Autophagy mediates anti-melanogenic activity of 3’-ODI in B16F1 melanoma cells. Biochem. Biophys. Res. Commun..

[88] Kalfalah F., Janke L., Schiavi A., Tigges J., Ix A., Ventura N., Boege F., Reinke H. (2016). Crosstalk of clock gene expression and autophagy in aging. Aging.

[89] Deruy E., Nassour J., Martin N., Vercamer C., Malaquin N., Bertout J., Chelli F., Pourtier A., Pluquet O., Abbadie C. (2014). Level of macroautophagy drives senescent keratinocytes into cell death or neoplastic evasion. Cell Death Dis..

[90] Kadono S., Manaka I., Kawashima M., Kobayashi T., Imokawa G. (2001). The role of the epidermal endothelin cascade in the hyperpigmentation mechanism of lentigo senilis. J. Investig. Dermatol..

[91] Hattori H., Kawashima M., Ichikawa Y., Imokawa G. (2004). The epidermal stem cell factor is over-expressed in lentigo senilis: Implication for the mechanism of hyperpigmentation. J. Investig. Dermatol..

[92] Choi W., Yin L., Smuda C., Batzer J., Hearing V.J., Kolbe L. (2017). Molecular and histological characterization of age spots. Exp. Dermatol..

[93] Imokawa G. (2019). Melanocyte Activation Mechanisms and Rational Therapeutic Treatments of Solar Lentigos. Int. J. Mol. Sci..

[94] Lee A.Y. (2015). Recent progress in melasma pathogenesis. Pigment. Cell Melanoma Res..

[95] Flori E., Mastrofrancesco A., Mosca S., Ottaviani M., Briganti S., Cardinali G., Filoni A., Cameli N., Zaccarini M., Zouboulis C.C. (2022). Sebocytes contribute to melasma onset. iScience.

[96] Nakama M., Murakami Y., Tanaka H., Nakata S. (2012). Decrease in nicotinamide adenine dinucleotide dehydrogenase is related to skin pigmentation. J. Cosmet. Dermatol..

[97] Okazaki M., Yoshimura K., Uchida G., Harii K. (2005). Correlation between age and the secretions of melanocyte-stimulating cytokines in cultured keratinocytes and fibroblasts. Br. J. Dermatol..

[98] Capasso S., Alessio N., Squillaro T., Di Bernardo G., Melone M.A., Cipollaro M., Peluso G., Galderisi U. (2015). Changes in autophagy, proteasome activity and metabolism to determine a specific signature for acute and chronic senescent mesenchymal stromal cells. Oncotarget.

[99] Ott C., König J., Höhn A., Jung T., Grune T. (2016). Macroautophagy is impaired in old murine brain tissue as well as in senescent human fibroblasts. Redox Biol..

[100] Kim N.H., Choi S.H., Yi N., Lee T.R., Lee A.Y. (2017). Arginase-2, a miR-1299 target, enhances pigmentation in melasma by reducing melanosome degradation via senescence-induced autophagy inhibition. Pigment. Cell Melanoma Res..

[101] Espósito A.C.C., de Souza N.P., Miot L.D.B., Miot H.A. (2021). Deficit in autophagy: A possible mechanism involved in melanocyte hyperfunction in melasma. Indian J. Dermatol. Venereol. Leprol..

[102] Ni C., Narzt M.S., Nagelreiter I.M., Zhang C.F., Larue L., Rossiter H., Grillari J., Tschachler E., Gruber F. (2016). Autophagy deficient melanocytes display a senescence associated secretory phenotype that includes oxidized lipid mediators. Int. J. Biochem. Cell Biol..

[103] Gupta A.K., Gover M.D., Nouri K., Taylor S. (2006). The treatment of melasma: A review of clinical trials. J. Am. Acad. Dermatol..

[104] Ando H., Kondoh H., Ichihashi M., Hearing V.J. (2007). Approaches to identify inhibitors of melanin biosynthesis via the quality control of tyrosinase. J. Investig. Dermatol..

[105] Ganesan A.K., Ho H., Bodemann B., Petersen S., Aruri J., Koshy S., Richardson Z., Le L.Q., Krasieva T., Roth M.G. (2008). Genome-wide siRNA-based functional genomics of pigmentation identifies novel genes and pathways that impact melanogenesis in human cells. PLoS Genet.

[106] Grill C., Bergsteinsdóttir K., Ogmundsdóttir M.H., Pogenberg V., Schepsky A., Wilmanns M., Pingault V., Steingrímsson E. (2013). MITF mutations associated with pigment deficiency syndromes and melanoma have different effects on protein function. Hum. Mol. Genet..

[107] Tse T.W., Hui E. (2013). Tranexamic acid: An important adjuvant in the treatment of melasma. J. Cosmet. Dermatol..

[108] Lee J.H., Park J.G., Lim S.H., Kim J.Y., Ahn K.Y., Kim M.Y., Park Y.M. (2006). Localized intradermal microinjection of tranexamic acid for treatment of melasma in Asian patients: A preliminary clinical trial. Dermatol. Surg..

[109] Li D., She Y., Li M., Liu J., Feng X. (2010). Tranexamic acid can treat ultraviolet radiation-induced pigmentation in guinea pigs. Eur. J. Dermatol..

[110] Kim M.S., Bang S.H., Kim J.H., Shin H.J., Choi J.H., Chang S.E. (2015). Tranexamic Acid Diminishes Laser-Induced Melanogenesis. Ann. Dermatol..

[111] Cho Y.H., Park J.E., Lim D.S., Lee J.S. (2017). Tranexamic acid inhibits melanogenesis by activating the autophagy system in cultured melanoma cells. J. Dermatol. Sci..

[112] Rubinsztein D.C., Gestwicki J.E., Murphy L.O., Klionsky D.J. (2007). Potential therapeutic applications of autophagy. Nat. Rev. Drug. Discov..

